# Comparison of the relative sensitivity of two dimensional personality models to the psychopathological symptoms: the section III DSM-5 maladaptive traits versus affective temperaments

**DOI:** 10.1186/s12888-022-04156-y

**Published:** 2022-07-27

**Authors:** Saeid Komasi, Azad Hemmati, Farzin Rezaei, Khaled Rahmani, Jouko Miettunen, Federico Amianto, Christopher J. Hopwood

**Affiliations:** 1 Student Research Committee, Kurdistan University of Medical Sciences, Sanandaj, Iran; 2 Neurosciences Research Center, Research Institute for Health Development, Kurdistan University of Medical Sciences, Sanandaj, Iran; 3 Department of Psychology, University of Kurdistan, Sanandaj, Iran; 4 Department of Psychiatry, Roozbeh Hospital, Tehran University of Medical Sciences, Tehran, Iran; 5 Liver and Digestive Research Center, Research Institute for Health Development, Kurdistan University of Medical Sciences, Sanandaj, Iran; 6 Center for Life Course Health Research, University of Oulu, & Medical Research Center Oulu, Oulu University Hospital and University of Oulu, Oulu, Finland; 7 Department of Neurosciences, Psychiatry Section, Regional Pilot Centre for Eating Disorders, University of Torino, Torino, Italy; 8 Department of Psychology, University of Zurich, Zurich, Switzerland

**Keywords:** Dimensional assessment, Mental symptoms, Personality disorder, Psychopathology, Temperament

## Abstract

**Background:**

The Personality Inventory for DSM-5 (PID-5) and Temperament Evaluation of Memphis, Pisa, Paris, and San Diego Autoquestionnaire (TEMPS-A) are tools designed for personality dispositions for mental health symptoms. The present study was conducted to compare these models in terms of their relative sensitivity to the symptoms of personality disorders (PDs) and non-personality disorders (NPDs).

**Methods:**

Subjects in this cross-sectional study were 1232 (805 female; 63.5%) community samples in western Iran. Data were collected using the PID-5, the TEMPS-A, the Symptom Checklist-90 (SCL-90-R), and the Personality Diagnostic Questionnaire (PDQ-4). Correlations and Regression models were used to examine associations between traits and symptoms.

**Results:**

Maladaptive traits assessed by the PID-5 were more strongly associated with PD symptoms, whereas affective temperaments measured by the TEMPS-A were more strongly associated with NPD symptoms.

**Conclusion:**

The present findings highlighted the practical utility of both the PID-5 and TEMPS-A indicating risk for psychopathology, but also suggest a distinction between PDs and NPDs in terms of underlying personality dispositions.

**Supplementary Information:**

The online version contains supplementary material available at 10.1186/s12888-022-04156-y.

## Background

Contemporary psychopathology research has now made it clear that individual differences in relatively stable dispositions predict risk for mental health symptoms, and that these individual differences can be organized within integrative, hierarchical frameworks. This point of view raises questions about the distinction between personality disorders (PDs) and other kinds of disorders, or non-PDs (NPDs; e.g., mood, anxiety, psychotic, or somatic disorders). One potential approach to determining whether there is a difference between these classes of psychopathology is to test whether there are different underlying trait dispositions. The main goal of this study was to test the relative specificity of a model of the trait risk factors for PDs and a model of trait risk factors for NPDs.

### Personality disorders

The proposal to replace the categorical approach to psychopathology with the dimensional approach in the fifth edition of the Diagnostic and Statistical Manual of Mental Disorders (DSM-5) was one of the most important and controversial changes [1]. Despite some challenges, the dimensional approach does not have many of the issues of the categorical approach [2] and has been widely welcomed for clinical diagnosis and application [3, 4]. Following the dimensional approach, one of the most important suggestions in the DSM-5 is the addition of an Alternative Model for Personality Disorders (AMPD) in the third section [1]. AMPD has been the subject of many studies over the last decade [5, 6]. According to the two criteria A and B proposed in this model, four personality disorders (PDs) including paranoid, schizoid, histrionic, and dependent were excluded from the list of ten previous diagnostic categories [1]. Criterion A expresses the intrapersonal (identity and self-direction) and interpersonal (empathy and intimacy) functions of the personality. This criterion in AMPD is a useful indicator for the general diagnosis of any personality disorder. Criterion B identifies 25 maladaptive personality traits that are organized within five major pathological domains, including negative affectivity, detachment, antagonism, disinhibition, and thought disorder or psychoticism [7]. These traits can be measured using the Personality Inventory for DSM-5 (PID-5). The PID-5 structure, which was developed via factor analytic methods [8], has recently been validated in many cultures and countries [9–12]. Recent reviews and meta-analyses support the validity and capability of this maladaptive trait model for diagnosing PDs [13, 14].

Although AMPD was initially proposed only for the diagnosis of PDs, subsequent research has suggested that traits can be used to organize all mental disorders [15]. For instance, the structure of the hierarchical taxonomy of psychopathology (HiTOP) includes maladaptive traits originally designed to indicate symptoms of personality disorders (PDs), along with the symptoms of other kinds of psychopathology (NPDs) [16, 17]. Subsequently, several studies have examined and confirmed the evidence-based structure and clinical and therapeutic application of HiTOP [18–20]. This implies some ambiguity in the distinction between PDs and NPDs, at least in terms of symptom covariance in cross-sectional data. Specifically, the HiTOP structure raises questions about whether PDs and non-PDs are actually different by implying that appears is that the same underlying dimensions can be used to describe variation. However, other theoretical work suggests important differences between PDs and non-PDs in terms of the relative specificity of risk factors related to each diagnostic category [21].

### Non-personality disorders

Indeed, some research suggests that the underlying sources for NPDs may differ from the dispositions for PDs, with particular attention having been given to affective temperaments [22, 23]. Temperaments are thought to be biologically driven factors that represent instinctive responses to the environment epigenetics [24–26]. Affective temperament is closely associated with vulnerability to the internalizing psychopathology and symptoms of some NPDs [23, 27, 28]. This is consistent with the coverage of NPDs by the internalizing spectrum in the HiTOP. Conversely, the HiTOP considers the symptoms of PDs mainly as lower-order factors in the spectra of externalizing, detachment, and thought disorder than internalizing spectrum [15]. Several review and meta-analytic studies have also pointed to the role of temperamental models in explaining the symptoms of NPDs [29–31]. The results of a comprehensive meta-analysis also showed that temperamental traits have a stronger relationship with NPDs than PDs [32].

Many temperament models were designed and developed under the concepts of Gray's theory of brain-behavioral systems, including the behavioral activation system, behavioral inhibition system, and fight and flight system [33]. The model of affective temperaments proposed by Akiskal et al. [24], is one of the theoretical frameworks that has led to extensive studies in recent decades. This model, which includes five premorbid dimensions of depressive, cyclothymic, hyperthymic, irritable, and anxious temperaments [25], is measured using the Temperament Evaluation of Memphis, Pisa, Paris, and San Diego Autoquestionnaire (TEMPS-A). Recent reports have shown the extensive and complex relationships between TEMPS-A temperaments and symptoms of some NPDs [34–37].

### Study aims

The emerging consensus in psychopathology research that a few underlying dimensions can account for the covariance structure of psychopathology has raised questions about whether there is an important difference between certain classes of psychopathology in other ways, including underlying etiological risk factors [38]. In this study, we focus on this question as it pertains to potential differences between PD and non-PD diagnoses. To improve the diagnostic process and remove ambiguous boundaries between diagnostic categories, as well as to facilitate clinical and therapeutic application, current hierarchical frameworks such as HiTOP attempt to introduce symptoms of PDs and NPDs as lower-order factors in some larger spectrums. While this may be a promising evolution, there is still insufficient empirical evidence to integrate these symptoms, even if they are indeed overlapping to a considerable degree.

The PID-5 was designed to measure maladaptive personality traits and TEMPS-A to assess temperamental predispositions for psychopathology. In this study, we tested the specificity of these models to the symptoms of PDs and NPDs. Evidence for specificity would support differences between PDs and NPDs in terms of underlying trait dispositions, whereas finding that both trait models predict PDs and NPDs similarly would challenge the distinction between these domains of psychopathology. A secondary goal of this study was to extend findings on this topic that have primarily come from Western (North American and European samples) to an Iranian sample.

## Methods

### Design and participants

Participants in this cross-sectional study were 1232 (805 female; 65.3% vs. 427 male; 34.7%) community members in the west of Iran (Kermanshah and Sanandaj cities) between April 2020 and August 2021. Quota sampling was performed from different population groups including college students, housewives (a woman whose main occupation is caring for her family, managing household affairs, and doing housework), employed and self-employed people, and retired and unemployed subjects. Following the quotas set for each group, the data collector referred to the institutions, academic centers, government and non-government organizations, and homes of housewives. The participation was voluntary without payment and after obtaining informed consent, those who met the inclusion criteria were asked to participate in the study. Inclusion criteria were 18 to 80 years old, no use of psychiatric medications or psychotherapy in the last two weeks, lack of current drug addiction or pharmacotherapy, and fluency in the Farsi language. Outliers and questionnaires with more than 10% of missing data were excluded from the study. The sample consisted of 1232 people (after deleting four outliers from the final analysis). The mean age and standard deviation of the subjects was 33.5 ± 11.1 years. Other demographic characteristics of the subjects were: 613 (48.8%) single and 619 (50.2%) married subjects; 493 (40%) people with a diploma or lower education and 739 (60%) people with a college degree; 318 (25.8%) housewives, 287 (23.3%) college student, 282 (22.9%) self-employed, 217 (17.6%) employed, 90 (7.3%) unemployed, and 38 (3.1%) retired people. To provide a better picture of the Iranian general population participating in the present study, Supplement 1 contains the data on the symptomology derived from standard T-scores of all SCL-90 and PDQ-4 subscales. To collect data, the study process was first explained to the subjects by an expert clinical psychologist. After obtaining informed consent to participate in the study, the subjects completed the demographic information form (gender, age, education level, job, and marital status). All participants answered the PID-5 (220 items), the short form of the TEMPS-A (35 items), the Revised Form of Symptom Checklist-90 (SCL-90-R; 90 items), and the Fourth Edition of the Personality Diagnostic Questionnaire (PDQ-4; 100 items).

### Variables measure

#### Personality Inventory for DSM-5 (PID-5)

This is a 220-item self-report inventory that was developed by Krueger et al., [39] to assess the five personality pathological domains and 25 facets according to criterion B of the AMPD proposed in DSM-5 Section-III. The domains (and their facets) include negative affectivity (emotional liability, anxiousness, and separation insecurity), detachment (withdrawal, anhedonia, and intimacy avoidance), antagonism (manipulativeness, deceitfulness, and grandiosity), disinhibition (irresponsibility, impulsivity, and distractibility), and thought disorder (unusual beliefs & experiences, eccentricity, and perceptual dysregulation). Other facets that are listed as components for more than one domain include attention-seeking, callousness, depressivity, hostility, perseveration, restricted affectivity, rigid perfectionism, risk-taking, submissiveness, and suspiciousness. Items response is based on a Likert scale ranging from zero to three [39]. Hemmati et al., [19] confirmed the validity and reliability of the Persian version using Cronbach's alphas (disinhibition: 0.89, detachment and negative affectivity: 0.93, and antagonism and thought disorder: 0.94). Also, Cronbach's alphas for the 25 trait facets were acceptable, ranging from 0.70 to 0.94. We used the domains score and their 15 lower-order facets for the current analysis. In the present study, Cronbach's alpha was .85 to .88 for the five domains and .94 to .95 for all facets.

#### Temperament Evaluation of Memphis, Pisa, Paris, and San Diego Autoquestionnaire (TEMPS-A)

The questionnaire was designed by Akiskal et al., [40] based on a semi-structured interview to assess affective temperaments. The original questionnaire has 110 items and the short form includes 39 items. The Persian version includes 35 items (Yes = 1/No = 0) in five subscales including depressive (8 items), cyclothymic (7 items) hyperthymic (8 items), irritable (6 items), and anxious (6 items) temperaments. Khalili et al. [41] confirmed the reliability (Cronbach's alpha ranging from .60 and .66 for the subscales) and validity of the Persian version of TEMPS-A in an Iranian sample. In the present study, Cronbach's alpha was between .52 and .82 for the five subscales.

#### Revised Form of Symptom Checklist-90 (SCL-90-R)

This test consists of 90 questions to assess the symptoms of mental disorders and separates healthy people from patients. The questionnaire was designed and revised by Derogatis et al., [42, 43] This questionnaire assesses nine clinical dimensions including somatization (12 items), obsessive–compulsive disorder (10 items), interpersonal sensitivity (9 items), depression (13 items), anxiety (10 items), hostility (6 items), phobic anxiety (7 items), paranoid ideation (6 items), and psychoticism (10 items). The answers to each question are graded based on a five-point Likert scale from no discomfort (zero points) to very severe discomfort (four points). Derogatis et al., [42, 43] showed that all dimensions of this checklist have construct and concurrent validity with the MMPI questionnaire. Also, Cronbach's alpha of all subscales in Iranian samples is reported between 0.75 to 0.92 [44, 45]. In the present study, Cronbach's alpha was .95 for the whole checklist and between .94 and .95 for all subscales.

#### Fourth Edition of the Personality Diagnostic Questionnaire (PDQ-4)

This 100-item self-report questionnaire was designed by Bagby and Farvolden [46] to diagnose symptoms of PDs. This tool evaluates 12 PDs including paranoid (7 items), schizoid (7 items), schizotypal (9 items), antisocial (7 items), borderline (9 items), narcissistic (9 items), histrionic (8 items), avoidant (7 items), dependent (8 items), obsessive–compulsive (8 items), depressive (7 items), and negativistic (7 items) PDs. The answers to the questions are yes (= 1) or no (= 0). This questionnaire has acceptable validity and its reliability has been reported between 0.56 to 0.84 using Cronbach's alpha for all subscales [46]. Cronbach's alpha of the subscales in Iranian samples is reported between 0.52 to 0.90 [47]. According to the diagnostic categories presented in section II DSM-5, two subscales of depressive and negativistic PDs were excluded from the present study. In the present study, Cronbach's alpha was .89 for the total scale and ranged from .87 and .88 for the ten subscales.

### Statistical analysis

In the first stage, the means and standard deviations of all variables and the Pearson correlations between PID-5 domains and TEMPS-A temperaments, and the symptoms of NPDs and PDs were reported. After observing a strong pattern of intercorrelations for both PD and non-PD measures, we conducted a conjoint Exploratory Factor Analysis (EFA) with maximum likelihood estimations on both sets of scales, the details of which can be seen in the results section. We concluded based on this analysis that a single variable could be used to conceptualize both PDs and non-PDs. We, therefore, used factor scores from these EFAs as DVs to construct 4 regression models, in which the domains of the PID-5 and TEMPS-A were alternatively entered as blocks to predict SCL-90 and PDQ-4 symptoms. We compared the change in R^2^ to determine how much additional variance each model was explaining in the outcome.

## Results

Table 1 contains the mean, standard deviation, and correlations between the PID-5 domains and TEMPS-A temperaments and the criterion variables. The results of this table show that all PID-5 maladaptive traits and TEMPS-A temperaments (except hyperthymic) are positively related to the SCL-90 and PDQ-4 symptoms (*p* < 0.05). The direction and significance of the relationship between hyperthymic temperament and the SCL-90 and PDQ-4 symptoms are scattered.

**Table 1:** The mean, standard deviation, and correlations between the maladaptive domains/affective temperaments and the symptoms of all NPDs and PDs

Variables (Mean ± SD)	Maladaptive Traits, PID-5^a^					Affective Temperaments, TEMPS-A^a^				
	NA (8.20 ± 4.00)	Det (7.76 ± 3.81)	Ant (6.18 ± 3.32)	Dis (6.31 ± 3.74)	TD (8.28 ± 5.71)	Dep (2.69 ± 2.37)	Cyc (3.33 ± 1.73)	Hyp (4.46 ± 1.92)	Irr (1.83 ± 1.74)	Anx (1.38 ± 1.44)
NPDs, SCL-90-R										
Somatization (12.72 ± 9.51)	.462	.425	.283	.455	.475	.502	.392	- .098	.551	.463
Obsessive–compulsive disorder (13.69 ± 7.86)	.564	.485	.242	.508	.466	.604	.477	- .159	.565	.413
Interpersonal sensitivity (11.07 ± 7.12)	.595	.524	.318	.553	.515	.643	.442	- .154	.606	.509
Depression (15.69 ± 11.26)	.618	.554	.260	.530	.476	.689	.457	- .225	.634	.475
Anxiety (9.94 ± 8.16)	.629	.511	.333	.557	.555	.642	.460	- .140	.685	.540
Hostility (6.89 ± 5.10)	.533	.418	.392	.533	.500	.562	.451	- .044*	.589	.509
Phobic anxiety (5.30 ± 5.47)	.524	.513	.388	.553	.571	.555	.318	- .098	.559	.554
Paranoid ideation (8.18 ± 4.99)	.537	.458	.362	.456	.497	.530	.445	- .021*	.523	.403
Psychoticism (8.16 ± 7.40)	.572	.554	.419	.609	.638	.611	.393	- .111	.604	.552
PDs, PDQ-4										
Paranoid (2.95 ± 1.88)	.430	.328	.337	.291	.353	.288	.310	.014*	.256	.200
Schizoid (2.14 ± 1.55)	.300	.544	.323	.331	.414	.300	.203	- .056 ^b^	.286	.270
Schizotypal (2.46 ± 1.87)	.417	.438	.442	.381	.604	.310	.273	.021*	.349	.342
Antisocial (1.54 ± 1.68)	.349	.340	.556	.525	.524	.333	.294	.091	.349	.421
Borderline (2.51 ± 2.09)	.583	.456	.432	.594	.537	.533	.436	- .083	.506	.445
Narcissistic (2.65 ± 1.90)	.350	.260	.562	.398	.468	.263	.297	.211	.263	.314
Histrionic (2.59 ± 1.77)	.446	.168	.494	.435	.424	.304	.354	.173	.270	.319
Avoidant (1.98 ± 1.69)	.479	.426	.260	.433	.379	.447	.272	- .154	.378	.346
Dependent (1.82 ± 1.87)	.530	.442	.394	.558	.477	.514	.291	- .126	.434	.433
Obsessive–compulsive (2.83 ± 1.72)	.398	.353	.329	.338	.382	.344	.323	.019*	.252	.197

*Note*. All columns contain Pearson correlation coefficients (r); * = Non-significant correlations; a = *P* value < 0.01 for all correlations; b = *P* value < 0.05

*Abbreviations*: *NA* Negative Affectivity, *Det* Detachment, *Ant* Antagonism, *Dis* Disinhibition, *TD* Thought Disorder, *Dep* Depressive, *Cyc* Cyclothymic, *Hyp* Hyperthymic, *Irr* Irritable, *Anx* Anxious, *PID-5* Personality Inventory for DSM-5, *TEMPS-A* Temperament Evaluation of Memphis, Pisa, Paris, and San Diego Autoquestionnaire, *SCL-90-R* Revised Form of Symptom Checklist-90, *PDQ-4* Fourth Edition of the Personality Diagnostic Questionnaire, *SD* Standard deviation

As described above, this table also suggests a high degree of non-specificity in the patterns across PD and NPD variables. For instance, correlations between SCL-90 psychoticism and PID-5 thought disorder are very high for all trait and temperament scales, rather than specific to those measuring thought disorder and emotional distress, respectively. Similar homogeneity in patterns was found for the PDQ-4 scales. Moreover, previous research suggests that each of these scales has issues related to discriminant validity [48, 49]. To test the degree to which the SCL-90 and PDQ-4 scales were measuring distinct disorders, we conducted EFA with maximum likelihood on both sets of scales. In both cases, we found only one eigenvalue > 1, strongly suggesting that, despite having scales designed to measure distinct forms of NPDs and PDs, respectively, they are really only providing one reliable general factor within each domain. To test whether these measures were able to distinguish between NPDs and PDs, we conducted a conjoint EFA with maximum likelihood estimation and found two factors with eigenvalues > 1 (9.24 and 2.78). We rotated these factors with Promax and present pattern coefficients in Table 2. All SCL-90 scales loaded on the first factor, whereas all PDQ-4 scales loaded on the second. All coefficients were quite strong (> .55) and cross-factor coefficients were all weak (|< .22|). We concluded that these measures could distinguish between NPDs and PDs, but not between different NPDs and PDs. We thus retained factor scores from the conjoint model to examine how the symptoms of NPDs and PDs are related to the PID-5 and TEMPS-A scales.

**Table 2:** Pattern Coefficients from a Conjoint Exploratory Factor Analysis of SCL-90 and PDQ-4 symptom scales

Variables	Non-PD symptoms	PD symptoms
SCL-90-R		
Somatization	.884	-.112
Obsessive–compulsive disorder	.904	-.056
Interpersonal Sensitivity	.911	.006
Depression	.921	-.032
Anxiety	.943	-.027
Hostility	.765	.074
Phobic Anxiety	.824	-.016
Paranoid Ideation	.729	.134
Psychoticism	.850	.050
PDQ-4		
Paranoid	-.035	.652
Schizoid	-.005	.592
Schizotypal	-.003	.712
Antisocial	-.027	.698
Borderline	.213	.629
Narcissistic	-.137	.798
Histrionic	-.052	.681
Avoidant	.133	.568
Dependent	.208	.534
Obsessive–compulsive	-.027	.658

*Note*. All columns contain Pearson correlation coefficients (r) between the variables and the latent factors

*Abbreviations*: *SCL-90-R* Revised Form of Symptom Checklist-90, *PDQ-4* Fourth Edition of the Personality Diagnostic Questionnaire, *PD* personality disorder

We used hierarchical regression to examine the specificity of maladaptive (PID-5) and temperament (TEMPS-A) traits for predicting NPDs and PDs. Results are shown in Table 3. When the TEMPS-A was entered in the first block, it had an R^2^ of .586 for NPDs and .368 for PDs, whereas when the PID-5 was entered in the first block, it had an R^2^ of .522 for NPDs and .530 for PDs. We also report Beta coefficients from each of the specific dimensions, although we caution that, due to multicollinearity, these coefficients may not be stable. As such, our main focus is on the relative change in R^2^ values for models with PDs as opposed to NPDs as the dependent variables. The TEMPS-A explained more variance above and beyond the PID-5 when predicting NPDs (change in R^2^ = .134) relative to PDs (change in *R*^2^ = .031). Conversely, the PID-5 explained more variance above and beyond the TEMPS-A when predicting PDs (change in *R*^2^ = .193) relative to NPDs (change in *R*^2^ = .070). Overall, these results both confirm that both sets of underlying dimensions are relevant to both PDs and NPDs, but also provide some support for the hypothesis that NPDs and PDs could be distinguished, both in the factor analysis of SCL-90 and PDQ-4 scales, as well as in the associations between the resulting factors and maladaptive traits as opposed to affective temperaments.

**Table 3:** Regression Models Comparing PID-5 and TEMPS-A as blocks to predict NPD and PD symptom factors

NPD symptom factors			PD symptom factors		
Block 1	Beta	*p*	Block 1	Beta	*p*
PID NA	.353	< .001	PID NA	.232	< .001
PID Det	.182	< .001	PID Det	.098	< .001
PID Ant	-.189	< .001	PID Ant	.227	< .001
PID Dis	.193	< .001	PID Dis	.078	.024
PID TD	.233	< .001	PID TD	.225	< .001
R^2^	.522	< .001	R^2^	.530	< .001
Block 2			Block 2		
PID NA	.165	< .001	PID NA	.150	< .001
PID Det	.102	< .001	PID Det	.105	< .001
PID Ant	-.150	< .001	PID Ant	.221	< .001
PID Dis	.062	.044	PID Dis	.025	.464
PID TD	.190	< .001	PID TD	.190	< .001
TEMPS Dep	.215	< .001	TEMPS Dep	.119	< .001
TEMPS Cyc	.104	< .001	TEMPS Cyc	.110	< .001
TEMPS Hyp	-.021	.291	TEMPS Hyp	.043	.052
TEMPS Irr	.232	< .001	TEMPS Irr	.027	.376
TEMPS Anx	.056	.025	TEMPS Anx	.022	.441
Change R^2^	.134	< .001	Change R^2^	.031	< .001
Block 1	Beta	*p*	Block 1	Beta	*p*
TEMPS Dep	.327	< .001	TEMPS Dep	.271	< .001
TEMPS Cyc	.152	< .001	TEMPS Cyc	.182	< .001
TEMPS Hyp	-.079	< .001	TEMPS Hyp	.068	.005
TEMPS Irr	.301	< .001	TEMPS Irr	.096	.008
TEMPS Anx	.117	< .001	TEMPS Anx	.196	< .001
R^2^	.586		R^2^	.368	< .001
Block 2			Block 2		
TEMPS Dep	.215	< .001	TEMPS Dep	.119	< .001
TEMPS Cyc	.104	< .001	TEMPS Cyc	.110	< .001
TEMPS Hyp	-.021	.291	TEMPS Hyp	.043	.052
TEMPS Irr	.232	< .001	TEMPS Irr	.027	.376
TEMPS Anx	.056	.025	TEMPS Anx	.022	.441
PID NA	.165	< .001	PID NA	.150	< .001
PID Det	.102	< .001	PID Det	.105	< .001
PID Ant	-.150	< .001	PID Ant	.221	< .001
PID Dis	.062	.044	PID Dis	.025	.464
PID TD	.190	< .001	PID TD	.190	< .001
Change R^2^	.070		Change R^2^	.193	< .001

*Note*. *PID-5* Personality Inventory for DSM-5, *TEMPS-A* Temperament Evaluation of Memphis, Pisa, Paris, and San Diego Auto questionnaire, *PD* Personality disorder, *NPD* Non-personality disorder, *NA* Negative Affectivity, *Det.* Detachment, *Ant.* Antagonism, *Dis.* Disinhibition, *TD* Thought Disorder. *Dep.* Depressive, *Cyc.* Cyclothymic, *Hyp.* Hyperthymic, *Irr* Irritable, *Anx* Anxious

Block 1 only includes either PID-5 domains or TEMPS-A temperaments, Block 2 includes both PID-5 domains and TEMPS-A temperaments

## Discussion

The main purpose of this study was to examine whether PDs and NPDs could be distinguished in terms of their associations with maladaptive traits designed to assess PD dispositions and affective temperaments designed to indicate risk for NPDs. Our results suggest that, although it is possible to synthesize individual differences in personality and general psychopathology into a single model [15], there is nevertheless a difference between PDs and NPDs. First, factor analyses of PD and NPD symptoms revealed two distinct factors. Second, these factors had differential relations with the PID-5 and TEMPS-A.

There were strong associations between all disposition measures and all symptom measures. These associations could be explained by a variety of factors, including method variance, the tendency for all kinds of psychopathology to covary, and discriminant validity issues in particular measures. The critical question, though, was whether, despite these various influences, we could find differences between PDs and NPDs. There is strong evidence that both types of disorders are influenced by genetic factors [50, 51], have similar patterns of stability [51], and can be organized using hierarchical trait models [15, 19]. This study provides evidence that, despite these similarities, differences between PDs and NPDs are evident in terms of the covariance of symptoms and associations with trait dispositions. This supports the previous distinction between “Axis I and II”, and challenges suggestions that all of the psychopathology can be integrated within a common structure.

The major question is, what is the distinction? One factor is that the TEMPS-A was designed primarily to indicate affective disorders [24, 25], whereas the PID-5 was designed to reorganize PD symptoms [36]. But what is the essential difference? The AMPD proposes that Criterion A or problems related to self and others is the distinguishing factor [7]. This proposal is similar to other theories that suggest that PDs can be distinguished as interpersonal disorders, whose core pathology has to do with how people navigate social relationships [52]. Imagining such a differentiating functional framework may help explain challenges in the social networks, including in the therapeutic alliance, that is a core marker of PDs. Our findings may confirm this because it was found that all domains except disinhibition are associated with symptoms of PD, whereas reciprocally this relationship was seen only for depressive and cyclothymic temperaments. PID-5, although highly capable of evaluating criterion B, probably also measures a significant amount of criterion A [53].

A secondary goal of this study was to extend findings on this topic that have primarily come from Western (North American and European samples) to an Iranian sample. A significant strength of this study was the use of a large sample that included participants from different segments of the Iranian population. It is important to extend findings on the structure and correlates of personality and psychopathology to non-Western samples. It would conversely be important to test whether these results would replicate and generalize in other cultural settings. Acceptable internal consistency of all measurement instruments and numerous extensive correlations between most of the variables under study confirmed the repetition and generalization of finding in the Iranian culture. Standard T-scores adapted from the present sample estimated the prevalence of symptoms of NPDs (total: ranging from 16.2 to 19.8%; severe: between 2.8 and 5.3%) and PDs (total: ranging from 14 to 21.4%; severe: between 1.9 and 7.1%) to be almost identical to those of other cultures [54–56]. Future work comparing these models in terms of measurement equivalence, stability, and other characteristics would be useful to inform how well findings translate across cultures.

## Limitations and future direction

To our knowledge, this study is pioneering research in comparing dimensional models of personality and psychopathology. However, one of the limitations of the present study is that some of the PID-5 facets load onto more than one domain that we did not include in the analysis. That is, like the PDQ-4, there is a large amount of shared variance in PID-5 traits that can affect the present results. It may even be possible to better explain the variance of symptoms of PDs by PID-5 than TEMPS-A as a result of the covariance and the high overlap between some of these variables. Morey et al., (2022) recently showed that this could be accounted for in part by criterion A [57]. Although Hopwood et al., [53] also noted the ability of PID-5 to estimate criterion A, our aim in the present study was not to examine personality functions.

It would also be important to extend these findings to clinical samples. Given our findings of discriminant issues, future work should use measures that can better distinguish varieties of psychopathology from one another. This would include different instruments, as well as methods other than self-report questionnaires that might enhance discriminant validity. As mentioned above, this highlights the need for clinical interviews to be used by experienced clinicians and researchers.

It should also not be overlooked that we have only measured the psychopathology traits and symptoms on the dimensional scale, not the established diagnosis of any PD or NPD. Response bias can occur in many areas of behavioral research that use self-reported data. Self-reported measures are biased due to demographic factors and may change over time [58]. Especially in assessing personality traits, it is likely that individuals have a general tendency toward positive responses, which in psychometrics refers to "constant error" [59]. Thus, we would not want to make diagnostic assumptions based solely on a few self-report inventories. In this case, it would be better to utilize clinical interviews and other research designs to further validate and expand upon the current findings. Although conducting face-to-face clinical interviews in large populations and extensive epidemiological studies is fraught with difficulties and complexities, using online formats to assess psychological symptoms and mental disorders can be helpful [60].

Finer-grained analyses of the maladaptive facets of Criterion B, as well as the role of Criterion A for distinguishing NPDs from PDs would usefully build upon the current study. Using the framework of HiTOP, and forthcoming measurement tools from that project would also be a useful future direction for examining the structure of psychopathology in general, as well as potential differences between NPDs and PDs within that structure. However, future studies should also examine the ability of other models to account for psychopathology and distinguish PDs from NPDs, such as those proposed by Cloninger [26] or Lara et al., [61]. Although this may be slightly different from the current research literature based on integrated psychopathology, it is necessary to examine the alliance or possible rupture of PDs from general psychopathology more carefully. Finally, future work should examine the alliance and clinical distinction of the PD/NPD for prognosis, treatment planning, and other aspects of clinical practice.

## Conclusions

The present findings highlighted the ability of both models measured using PID-5 and TEMPS-A in explaining the symptoms of personality and general psychopathology. However, these results also suggest some differentiation: the PID-5 was a more sensitive tool for assessing personality pathology while TEMPS-A was more useful for determining the severity of symptoms of other disorders. The different capabilities of these two models were indicated by exploratory factor analysis and hierarchical regression models. The results indicated a common root for TEMPS-A temperaments and the symptoms of NPDs versus PID-5 facets and the symptoms of PDs. In general, the findings support the different theoretical and practical structures of the two models measured using PID-5 and TEMPS-A. Future studies may examine the validation of the integrated model of PID-5 domains/facets and other temperamental models in explaining general psychopathology.

## Supplementary Information

**Additional file 1: Supplementary 1**. The data on the symptomology derived from standard T-scores of all SCL-90 and PDQ-4 subscales.

## Data Availability

The current study data are available on reasonable request to S.K., S_komasi63@yahoo.com.
