# Supplementary material for: Comparison of the relative sensitivity of two dimensional personality models to the psychopathological symptoms: the section III DSM-5 maladaptive traits versus affective temperaments
Source: BMC Psychiatry. 2022 Jul 27;22:503. doi: 10.1186/s12888-022-04156-y (PMC9327203; doi:10.1186/s12888-022-04156-y)
Supplement: Supplementary file 1 — Additional file 1: Supplementary 1. The data on the symptomology derived from standard T-scores of all SCL-90 and PDQ-4 subscales. [file 12888_2022_4156_MOESM1_ESM.doc]

**Supplementary 1.** The data on the symptomology derived from standard T-scores of all SCL-90 and PDQ-4 subscales

| Scale | 60 ≤ T score < 70 (%) | 70 ≤ T score (%) | Cumulative (%) |
| --- | --- | --- | --- |
| SCL-90-R (total) | 17.1 | 2.8 | 19.9 |
| Somatization | 14.1 | 3.7 | 17.8 |
| Obsessive-compulsive disorder | 13.2 | 3.0 | 16.2 |
| Interpersonal Sensitivity | 14.2 | 2.8 | 17.0 |
| Depression | 14.1 | 2.8 | 16.9 |
| Anxiety | 14.2 | 3.0 | 17.2 |
| Hostility | 16.3 | 3.5 | 19.8 |
| Phobic Anxiety | 14.9 | 4.9 | 19.8 |
| Paranoid Ideation | 13.4 | 2.9 | 16.3 |
| Psychoticism | 13.1 | 5.3 | 18.4 |
| PDQ-4 (total) | 15.8 | 2.2 | 18.0 |
| Paranoid | 18.3 | 3.1 | 21.4 |
| Schizoid | 17.0 | 2.5 | 19.5 |
| Schizotypal | 12.7 | 2.5 | 15.2 |
| Antisocial | 6.9 | 7.1 | 14.0 |
| Borderline | 15.3 | 4.4 | 19.7 |
| Narcissistic | 14.0 | 3.1 | 17.1 |
| Histrionic | 13.4 | 1.9 | 15.3 |
| Avoidant | 14.6 | 3.6 | 18.2 |
| Dependent | 13.7 | 4.7 | 18.4 |
| Obsessive-compulsive | 14.8 | 1.9 | 16.7 |
